# Alterations of Gut Microbiota in Patients With Intestinal Tuberculosis That Different From Crohn’s Disease

**DOI:** 10.3389/fbioe.2021.673691

**Published:** 2021-07-06

**Authors:** Cong He, Huan Wang, Chen Yu, Chao Peng, Xu Shu, Wangdi Liao, Zhenhua Zhu

**Affiliations:** ^1^Department of Gastroenterology, The First Affiliated Hospital of Nanchang University, Nanchang, China; ^2^Department of Radiology, The First Affiliated Hospital of Nanchang University, Nanchang, China

**Keywords:** intestinal tuberculosis, Crohn’s disease, gut microbiota, 16S rRNA sequencing, short-chain fatty acids

## Abstract

Intestinal tuberculosis (ITB) and Crohn’s disease (CD) are chronic inflammatory bowel disorders that are associated with dysregulated mucosal immunity. The gut microbiota plays an important role in the regulation of host immunity and inflammatory response. Although mounting evidence has linked CD with the dysbiosis of gut microbiota, the characteristic profiles of mucosal bacteria in ITB remain unclear. The aim of this study was to assess the alterations of the gut microbiota in ITB and compare the microbial structure of ITB with CD. A total of 71 mucosal samples were collected from patients with ITB, CD, and healthy controls (HC), and then, 16S rRNA gene sequencing was performed. The overall composition of gut microbiota in ITB was strikingly different from HC, with the dominance of Proteobacteria and reduction of Firmicutes. Of note, the short-chain fatty acids (SCFAs)-producing bacteria such as *Faecalibacterium*, *Roseburia*, and *Ruminococcus* were decreased in ITB relative to HC, while *Klebsiella* and *Pseudomonas* were enriched. Multiple predictive functional modules were altered in ITB, including the over-representation of lipopolysaccharide biosynthesis, bacterial invasion of epithelial cells, and pathogenic *Escherichia coli* infection that can promote inflammation. Additionally, the microbial structure in CD was distinctly different from ITB, characterized by lower alpha diversity and increased abundance of *Bacteroides*, *Faecalibacterium*, *Collinsella*, and *Klebsiella*. These four bacterial markers distinguished ITB from CD with an area under the curve of 97.6%. This study established the compositional and functional perturbation of the gut microbiome in ITB and suggested the potential for using gut microbiota as biomarkers to differentiate ITB from CD.

## Introduction

Tuberculosis (TB), caused by the bacillus *Mycobacterium tuberculosis*, remains a public health problem and is one of the top 10 causes of death worldwide. About a quarter of the global population is infected with *M. tuberculosis*, and China is one of the 22 countries identified as having a high TB burden (World Health Organization, 2020). TB may involve any part of the body, including the lung, the primarily affected organ, and the gastrointestinal tract. Intestinal TB (ITB) is a chronic intestinal disease with non-specific clinical manifestation and endoscopic features, which were similar to Crohn’s disease (CD; He Y. et al., 2019). However, the therapeutic strategies between ITB and CD are completely different. ITB patients were treated with antituberculosis medication, while CD patients were administered immunosuppressive agents. The misdiagnosis between the two diseases would either miss the best time for treatment or even worsen the condition of the patient. As the current differential diagnostic methods are complicated and time-consuming, it is urgent to seek a convenient and efficient tool to help distinguish the two diseases in clinical practice.

With the development of high-throughput sequencing and bioinformatic analysis, there is mounting evidence that suggests the critical role of the gut microbiome in human health and disease (Feng et al., 2018). The dysbiosis of gut microbiota has been demonstrated in multiple diseases, including colorectal cancer, hepatocellular carcinoma, and inflammatory bowel disease, and the different microbial features showed the potential of disease prediction with high accuracy (Eck et al., 2017; Ren et al., 2019; Thomas et al., 2019). Several studies reported that the composition and function of gut microbiota in patients with CD were significantly different from the healthy subjects, and some of the differential microbes could classify patients by disease state with the AUC of 0.84 (He et al., 2017; Douglas et al., 2018). Our previous study also found that the mucosa-associated gut microbiota changed remarkably after the induction of remission in active CD patients, which indicates the potential ability of microbiota as a modality for response prediction (He C. et al., 2019). While there have been some studies showing the gut microbiome signatures in pulmonary tuberculosis patients, the microbial profiles of ITB remain largely unclear (Luo et al., 2017; Hu et al., 2019).

To date, with the increasing incidence of CD and a heavy burden of TB at the same time, it is of vital importance to differentiate CD and ITB, as the misdiagnosis may lead to fatal outcomes. In this study, we investigated the differences in the composition and function of the gut microbiota between ITB and healthy subjects. Furthermore, we compared the microbial communities of ITB with CD to explore the robust bacterial markers for disease discrimination. Our findings help define the gut dysbiosis of ITB and offer a novel insight into a microbiota-based model that can assist in clinical diagnosis.

## Materials and Methods

### Study Cohort and Sample Collection

This study was approved by the institutional review boards of The First Affiliated Hospital of Nanchang University. Written informed consent was obtained from all the subjects involved in the study. A total of 22 patients, including 6 with ITB and 16 with active CD, were recruited to the study cohort from the Department of Gastroenterology, The First Affiliated Hospital of Nanchang University, China. The diagnosis of ITB and CD was based on clinical, laboratorial, endoscopic, radiologic, and histological findings as previously described (He Y. et al., 2019). Inclusion criteria were newly diagnosed patients without the use of antibiotics, probiotics, and prebiotics for at least 1 month. Four age- and gender-matched healthy subjects without previous history of chronic disease and any drug usage were also enrolled. Seventy-one biopsy samples were collected from the ileum, ascending colon, and descending colon in each participant, although seven mucosal samples were failed for sequencing because of the small size and host contamination. All the samples were obtained during colonoscopy and frozen immediately at −80°C until DNA extraction.

### DNA Extraction and PCR Amplification

Total bacterial DNA was extracted from mucosal samples using the HiPure Stool DNA Kit (Magen, Guangzhou, China) according to the instructions of the manufacturer. The quality and concentration of DNA were detected using NanoDrop 2000C spectrophotometer (Thermo Fisher Scientific, Waltham, MA, United States). The DNA integrity was evaluated by 1% agarose gel electrophoresis. The V3–V4 regions of the 16S rRNA gene were amplified by PCR using primers 341F 5′-CCTACGGGNGGCWGCAG-3′ and 806R 5′-GGACTACHVGGGTATCTAAT-3′. Genomic DNA was initially denatured at 95°C for 2 min, followed by 27 cycles consisting of denaturation at 98°C for 10 s, annealing at 62°C for 30 s, elongation at 68°C for 30 s, and a final extension step at 68°C for 10 min. PCRs were performed in triplicate, 50 μl of a mixture containing 5 μl of 10× KOD buffer, 5 μl of 2.5 mM dNTPs, 1.5 μl of 5 μM primer, 1 μl of KOD polymerase, and 100 ng of template DNA.

### 16S rRNA Gene Sequencing

The PCR amplicons were extracted from 2% agarose gels and purified using the AxyPrep DNA Gel Extraction Kit (Axygen Biosciences, Union City, CA, United States) according to the protocols of the manufacturer, and they were quantified using the QuantiFluor-ST (Promega, Madison, WI, United States). Purified amplicons were pooled in equimolar quantities, and a sequencing library was constructed according to the official details of the Illumina. Subsequently, next-generation sequencing was performed using the Illumina Hiseq 2500 platform (Illumina, San Diego, CA, United States) by Genedenovo Biotechnology Co., Ltd. (Guangzhou, China) with 2 × 250 bp paired-end reads.

### Bioinformatics Analysis

Raw reads were filtered using FASTP (version 0.18.0): (1) remove reads containing more than 10% of unknown nucleotides and (2) remove reads containing less than 50% of bases with quality (Q-value) > 20 (Chen et al., 2018). Paired and clean reads were merged as raw tags using FLASH (version 1.2.11) with a minimum overlap of 10 bp and a mismatch error rate of 2% (Magoc and Salzberg, 2011). High-quality clean tags were obtained by QIIME (version 1.9.1) pipeline, and then, the chimera checking was performed based on the reference^1^ using UCHIME algorithm.^2^ The effective tags were clustered into operational taxonomic units (OTUs) with similarity ≥97% using UPARSE pipeline (Edgar, 2013). The tag sequence with the highest abundance was selected as a representative sequence. The representative OTUs were classified into organisms by a naive Bayesian model using the RDP classifier (version 2.2) based on the SILVA database (version 132) with the confidence threshold value of 0.8 (Wang et al., 2007).

The abundance statistics of each taxonomy was visualized using Krona (version 2.6) (Ondov et al., 2011). The stacked bar plot of the community composition was visualized in R project ggplot2 package (version 2.2.1). The alpha diversity indexes including Sobs, Shannon, and Chao1 were calculated in QIIME (Thukral, 2017). The beta diversity was estimated by the weighted UniFrac and Bray–Curtis distances and visualized with principal coordinates analysis (PCoA) in R project Vegan package (version 2.5.3). Species comparison between groups was performed by Welch’s *t*-test, and biomarker features were screened by randomForest package (version 4.5.12) in R. The KEGG pathway analysis of the OTUs was inferred using PICRUSt (version 2.1.4) to illustrate the predictive functional profiling of microbial communities (Langille et al., 2013).

### Statistical Analysis

Statistical analyses of demographic characteristics were performed using a one-way ANOVA for age and body mass index (BMI) and using chi-square test with Statistical Product and Service Solutions (SPSS) 20.0 (IBM) for gender. The comparison of alpha diversity indexes was calculated by Welch’s *t*-test. Statistical analysis of beta diversity between different groups was conducted by Adonis test. *P*-value <0.05 was considered as statistically significant. Bioinformatic analysis was performed using Omicsmart, a dynamic real-time interactive online platform for data analysis.^3^

## Results

Demographic characteristics of the subjects included in this study were shown in Supplementary Table 1. There was no significant difference of age, gender, and BMI among patients with CD, ITB, and healthy controls (HC). In order to get a comprehensive profile of the gut microbiota, mucosal samples were collected from three intestinal segments of each subject, including ileum, ascending colon, and descending colon, and then, 16S rRNA gene sequencing was performed. A total of 8,769,199 raw sequences were obtained. After quality filtering and binning, 8,048,679 sequences were retained for further analysis with an average of 113,361 sequences per sample (range 28,794–224,414 sequences/sample).

### The Microbial Features in ITB Compared With HC

**FIGURE 1:**
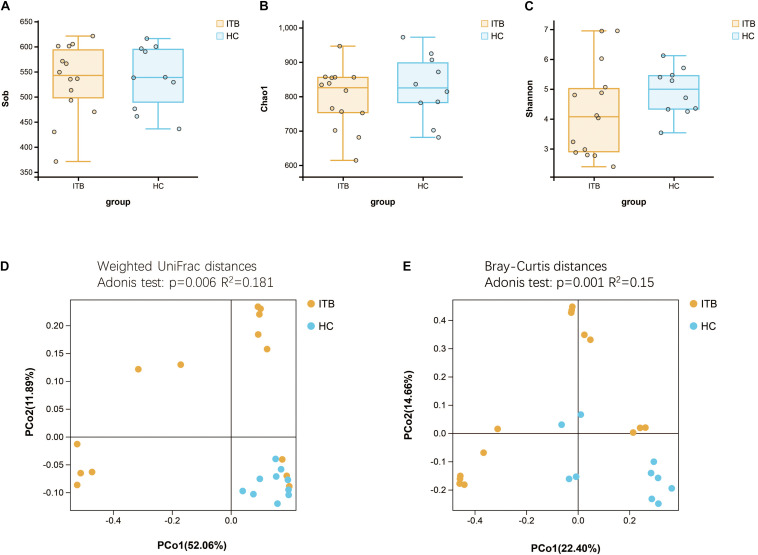
Comparison of the alpha and beta diversity of the gut microbiota between patients with ITB and HC. There was no significant difference in bacterial alpha diversity, as measured by Sob Index **(A)**, Chao1 Index **(B)**, and Shannon Index **(C)**, between ITB and HC. PCoA based on weighted UniFrac distances **(D)** and Bray–Curtis distances **(E)** showed that the overall microbiota structure was different between ITB and HC. ITB, intestinal tuberculosis; HC, healthy controls; Sob, number of observed operational taxonomic units; PCoA, principal coordinate analysis.

Microbial alpha diversity analysis revealed that the indexes including observed species (Sob), Chao1, and Shannon were not significantly different between ITB and HC (Figures 1A–C). We next assessed the dissimilarities between ITB and HC using the Bray–Curtis dissimilarity and weighted UniFrac distance metrics to evaluate the overall differences in beta diversity. The PCoA showed that samples of ITB clustered separately from those of HC (Figures 1D,E). The *p*-values obtained using the Adonis test for both distances were significant (*p* < 0.01), and the *R*^2^ values were indicative of remarkable variance between ITB and HC.

**FIGURE 2:**
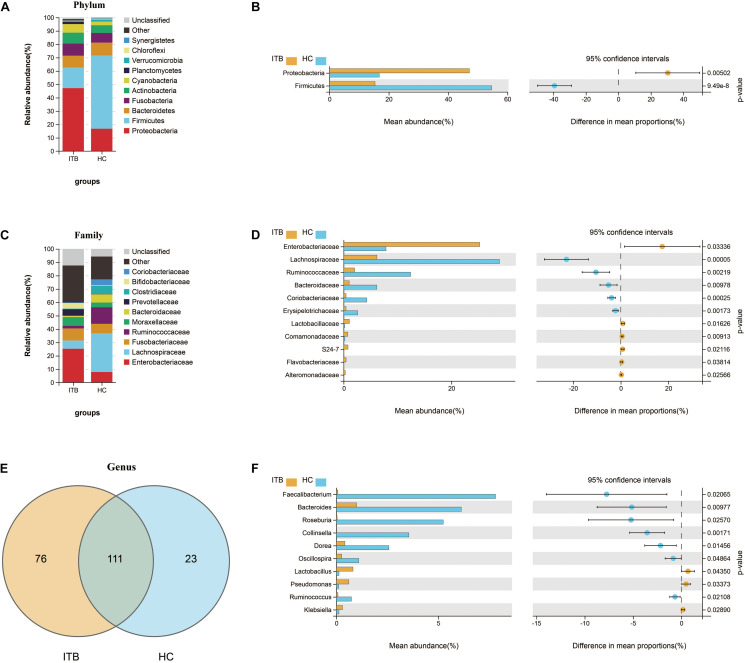
Variations of gut microbiota composition in ITB versus HC. Relative proportions of the top 10 most abundant bacteria at the phylum **(A)** and family **(C)** level in ITB and HC. The differentially abundant taxa between ITB and HC are identified at the phylum **(B)**, family **(D)**, and genus **(F)** level using Welch’s *t*-test. **(E)** Venn diagram showed the shared and unique bacterial genera between these two groups. ITB, intestinal tuberculosis; HC, healthy controls.

To investigate the specific changes of microbiota in samples of ITB, we assessed the relative abundance of taxa in ITB and HC. As shown in Figure 2A, the Firmicutes, Bacteroidetes, and Proteobacteria were the top three most abundant phyla, with differential distribution between ITB and HC. While Firmicutes were highly abundant in HC (54.7%), patients with ITB were dominated by Proteobacteria (47.2%) with a lower abundance of Firmicutes (15.4%) (Figure 2B). At the family level, the most abundant taxa included Enterobacteriaceae, Lachnospiraceae, Fusobacteriaceae, and Ruminococcaceae. Compared with HC, the relative abundance of Enterobacteriaceae belonging to Proteobacteria was higher in ITB, whereas Lachnospiraceae and Ruminococcaceae were prominently decreased which may lead to the reduction of Firmicutes (Figures 2C,D). There were 76 and 23 genera that were uniquely found in ITB and HC, respectively, while 111 genera were in common (Figure 2E and Supplementary Table 2). Of the 111 genera, we observed 10 bacterial taxa that displayed different abundance between ITB and HC (Figure 2F). Three genera including *Lactobacillus*, *Pseudomonas*, and *Klebsiella* were over-represented in ITB compared with HC. Conversely, seven genera (*Faecalibacterium*, *Bacteroides*, *Roseburia*, *Collinsella*, *Dorea*, *Oscillospira*, and *Ruminococcus*) were significantly decreased in ITB. Although *M. tuberculosis* was not detected in ITB at the species level, the genus *Mycobacterium* was more abundant in ITB compared with HC (Metastats analysis, *p* = 0.014). To assess the diagnostic value of gut microbial markers for ITB, we used genus information to construct a random forest classifier model between ITB and HC. Two specific importance measures including the mean decrease accuracy and the mean decrease in Gini were performed, and the top three most important genera in common (*Faecalibacterium*, *Roseburia*, and *Collinsella*) were chosen for subsequent analysis (Supplementary Figures 1A,B). The testing result showed that these three features were able to differentiate patients with ITB from HC with high accuracy (AUC = 1, 95% CI 1–1, Supplementary Figure 1C).

**FIGURE 3:**
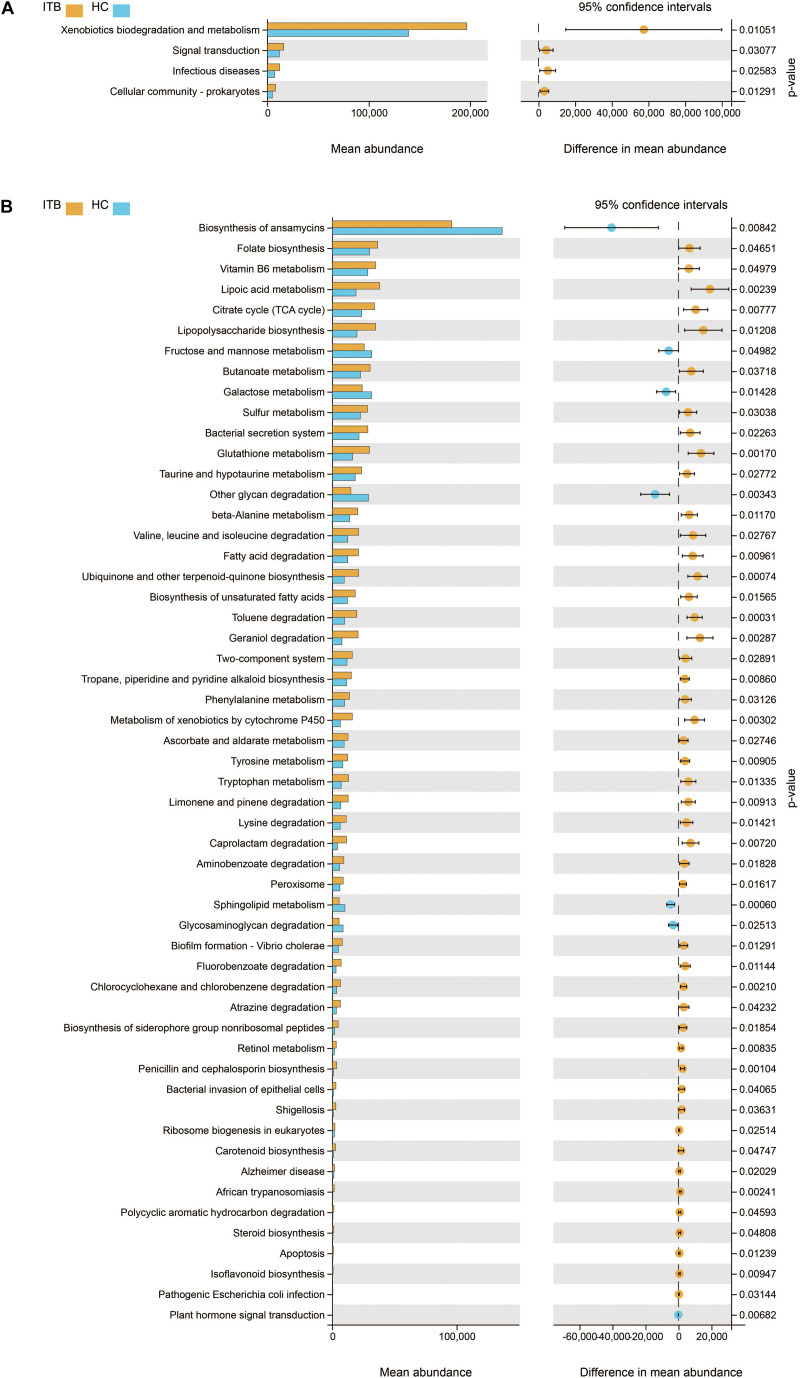
Comparison in the relative abundance of PICRUSt-generated functional profile of gut microbiota between ITB and HC. Panels **(A,B)** represent the KEGG pathways that are differentially abundant between these two groups at level 2 and level 3, respectively (Welch’s *t*-test). ITB, intestinal tuberculosis; HC, healthy controls; KEGG, Kyoto Encyclopedia of Genes and Genomes.

Furthermore, we used PICRUSt to infer the metagenome functional content based on the microbial community profiles obtained from the 16S rRNA gene sequences. The functional changes in ITB microbiomes included significantly increased representation of predicted KEGG pathways of level 2 involved in xenobiotics biodegradation and metabolism, signal transduction, infectious diseases (Figure 3A). The level 3 KEGG pathway data indicated that lipopolysaccharide biosynthesis, bacterial secretion system, bacterial invasion of epithelial cells, shigellosis, and pathogenic *Escherichia coli* infection were enriched in ITB compared with HC. In contrast, the gut microbiome of HC was characterized by over-representation of physiological pathways, including fructose and mannose metabolism, galactose metabolism, other glycan degradation, sphingolipid metabolism, and glycosaminoglycan degradation (Figure 3B).

### The Alterations of Gut Microbiota in Active CD in Comparison With HC

**FIGURE 4:**
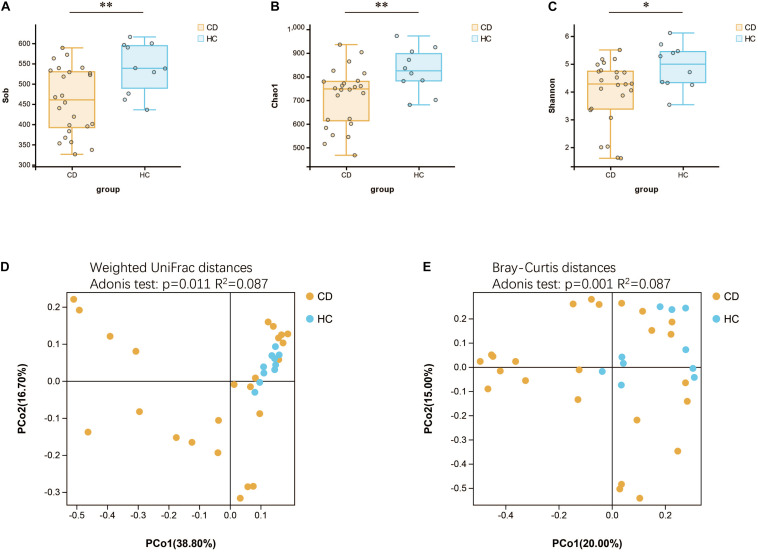
Changes of gut microbial biodiversity in patients with CD as compared with HC. Alpha diversity, as illustrated by Sob Index **(A)**, Chao1 Index **(B)**, and Shannon Index **(C)**, was significantly reduced in CD (Welch’s *t*-test). PCoA plots of weighted UniFrac distances **(D)** and Bray–Curtis distances **(E)** showed the beta diversity of the gut microbial communities in CD and HC, and a significant separation between these two groups has been found. CD, Crohn’s disease; HC, healthy controls; Sob, number of observed operational taxonomic units; PCoA, principal coordinate analysis. *p < 0.05, **p < 0.01.

Initially, the gut microbiota richness, as measured by Sob, Shannon, and Chao1 indexes, was reduced in patients with active CD as compared with HC (Figures 4A–C). Then, we sought to explore whether the overall bacterial phenotypes of CD and HC were different. Beta diversity was calculated using both the weighted UniFrac distances and Bray–Curtis distances and visualized in PCoA plots. The total diversity captured by the top two principal coordinates was 55.5 and 35% for the weighted UniFrac and Bray–Curtis distances, respectively. The microbiota composition of active CD was distinctly different from that of HC (Adonis test, *p* = 0.011, *R*^2^ = 0.087 for weighted distances, and *p* = 0.001, *R*^2^ = 0.087 for Bray–Curtis distances, Figures 4D,E).

**FIGURE 5:**
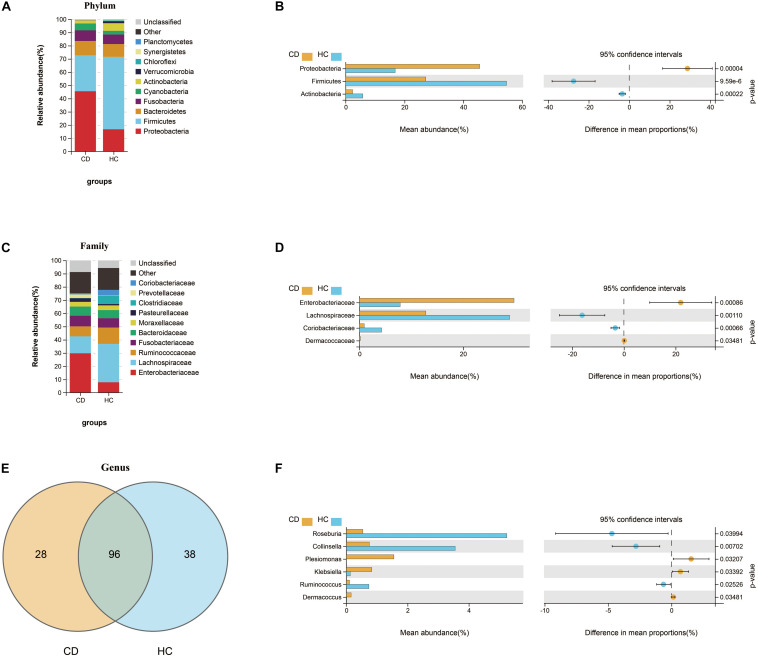
Compositional alterations of gut microbiota in CD versus HC. The stacked bar plots showed the relative proportion of the top 10 most abundant bacterial phyla **(A)** and families **(C)** in CD and HC. Comparison of the relative abundance of gut microbiota between CD and HC demonstrated differences in phyla **(B)**, families **(D)**, and genera **(F)** using Welch’s *t*-test shown as mean abundance (%) and difference in mean proportions (%) with 95% confidence intervals. **(E)** Venn diagram represented the shared and unique genera between CD and HC. CD, Crohn’s disease; HC, healthy controls.

Next, we investigated the compositional differences between active CD and HC at three microbial levels. Interestingly, patients with CD harbored increased abundance of Proteobacteria (45.6%) and decreased abundance of Firmicutes (27.2%), the changes of which were quite similar to ITB as compared with HC (Figures 5A,B). At the family level, the relative abundance of Enterobacteriaceae was upregulated in CD compared with HC, while Lachnospiraceae and Coriobacteriaceae were downregulated (Figures 5C,D). A total of 162 genera were identified and 96 genera were shared in both CD and HC (Figure 5E). Of these, 28 genera were unique to CD and 38 genera were unique to HC (Supplementary Table 3). Relative to HC, patients with active CD displayed a higher abundance of *Klebsiella*, *Plesiomonas*, and *Dermacoccus* and a lower abundance of *Roseburia*, *Collinsella*, and *Ruminococcus* (Figure 5F). To explore the diagnostic value of gut microbiota for CD, we constructed a random forest classifier model to distinguish CD patients from HC. Three genera including *Collinsella*, *Roseburia*, and *Haemophilus* were finally selected based on both the mean decrease Gini and the mean decrease accuracy measures (Supplementary Figures 2A,B). We applied the receiver operating characteristic (ROC) curve and found that the 3-genera set could separate CD from HC with an AUC of 87.5% (95% CI 0.752–0.998) (Supplementary Figure 2C).

**FIGURE 6:**
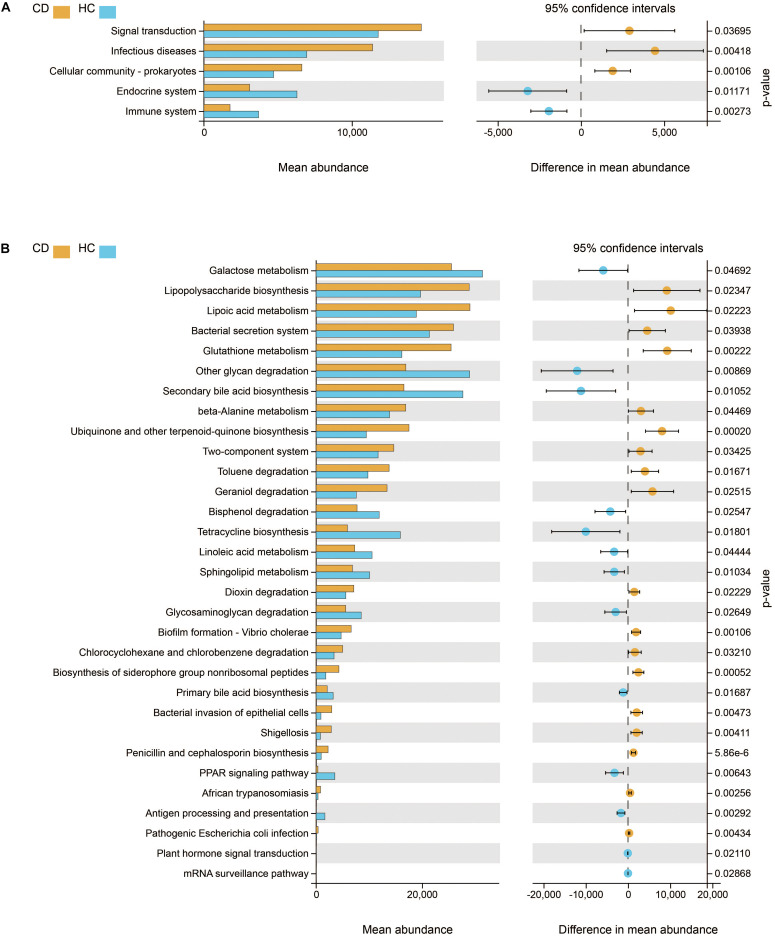
Functional features of gut microbiota in CD. The functional composition of gut microbiota based on PICRUSt prediction was compared between CD and HC using Welch’s *t*-test. **(A)** KEGG pathway at level 2 and **(B)** KEGG pathway at level 3. CD, Crohn’s disease; HC, healthy controls; KEGG, Kyoto Encyclopedia of Genes and Genomes.

We further studied the functional changes of microbial communities between CD and HC using PICRUSt. At KEGG level 2, we found that signal transduction and infectious diseases were enriched in CD compared with HC, while the endocrine system and immune system were inactivated (Figure 6A). Of note, functional pathways at level 3 including lipopolysaccharide biosynthesis, bacterial secretion system, bacterial invasion of epithelial cells, shigellosis, and pathogenic *E. coli* infection were much abundant in CD. In contrast, the physiological modules such as galactose metabolism, other glycan degradation, secondary bile acid biosynthesis, and linoleic acid metabolism were depleted in CD (Figure 6B).

### The Distinct Microbial Characteristics to Differentiate Between ITB and CD

**FIGURE 7:**
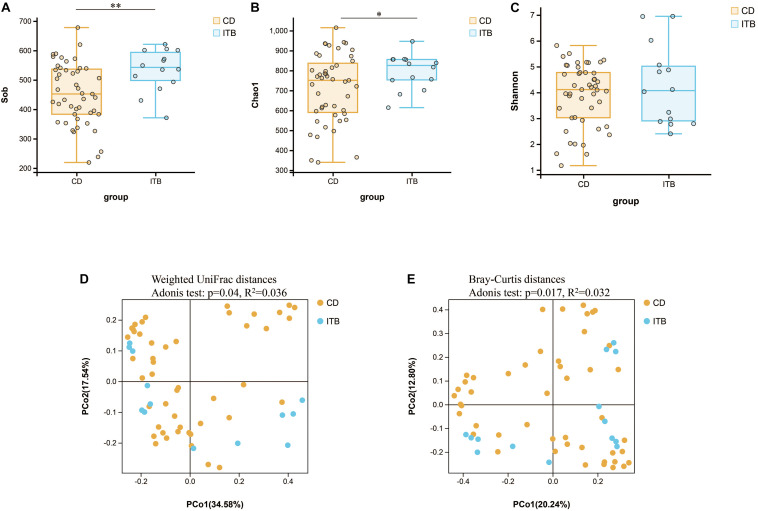
Variation of microbial diversity and community structure between ITB and CD. Alpha diversity analysis demonstrated that Sob **(A)** and Chao1 **(B)** were lower in CD than ITB, while no significant difference was observed in Shannon **(C)**. Beta diversity analysis indicated by PCoA plots of Weighted UniFrac distances **(D)** and Bray–Curtis distances **(E)** depicted the distinct clustering between ITB and CD. ITB, intestinal tuberculosis; CD, Crohn’s disease; Sob, number of observed operational taxonomic units; PCoA, principal coordinate analysis. *p < 0.05, **p < 0.01.

Despite the growing body of studies depicting the gut microbiome profiling in patients with CD, there is a paucity of literature exploring whether the structure of gut microbiota is different between CD and ITB. First, the analysis of alpha diversity revealed that both the richness and diversity as calculated in Sob and Chao1 were lower in CD compared with ITB, while no significant difference was observed in Shannon Index (Figures 7A–C). The analysis of beta diversity as calculated on the Bray–Curtis dissimilarity and weighted UniFrac distances showed the mucosal-associated microbial community of ITB samples apart from that of CD (Figures 7D,E), although the overall compositional difference between ITB and CD was not as striking as the distinction between either disease and HC.

**FIGURE 8:**
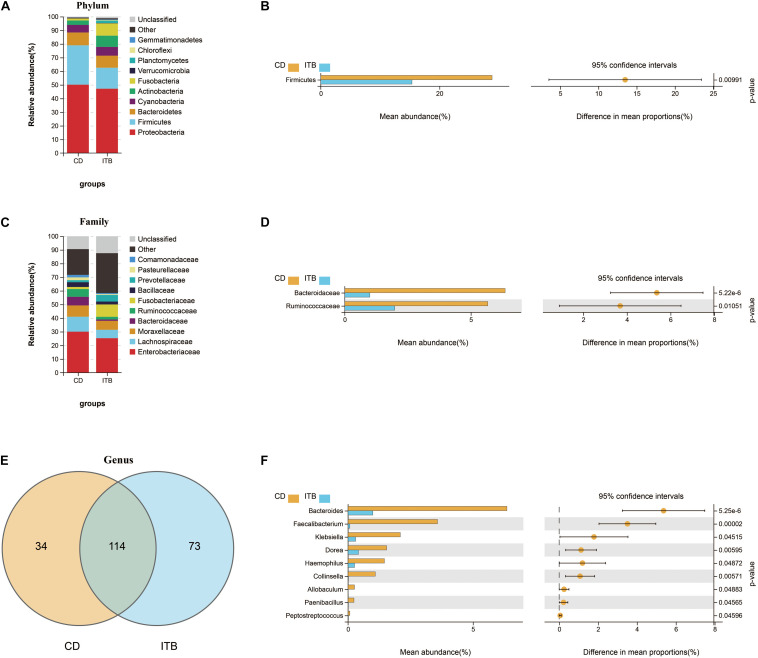
Dissimilarity analysis of gut microbiota composition between ITB and CD. The distinct distribution of the top 10 most abundant taxa in different groups at phylum **(A)** and family **(C)** level. Welch’s *t*-test was performed to identify the significantly changed phyla **(B)**, families **(D)**, and genera **(F)** between these two groups. **(E)** The shared/unique genera between ITB and CD were shown by Venn diagram.

**FIGURE 9:**
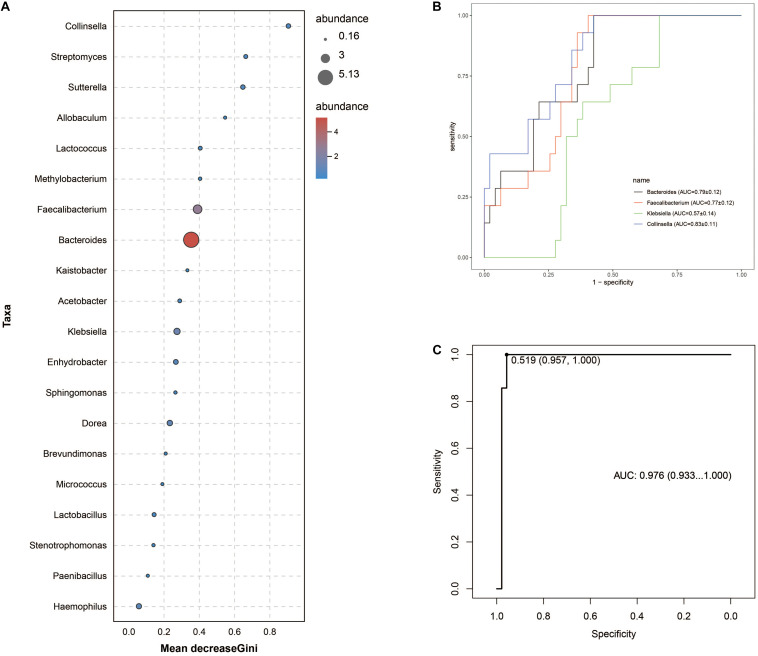
Gut microbiota biomarkers can be used to discriminate ITB from CD using random forest models. **(A)** The top 20 genera were detected for the classification between ITB and CD. Four biomarker taxa were identified based on mean decrease Gini Index and relative abundance. **(B)** The individual bacterial candidate could discriminate the two groups with AUC ranging from 0.57 to 0.83. **(C)** The combination of these four genera yielded more robust diagnostic performance over that of separate genera.

Intergroup comparisons of taxonomic profiles revealed that samples from ITB and CD exhibited alterations in the abundance of several taxa. At the phylum level, the relative abundance of Firmicutes was lower in ITB compared with CD (Figures 8A,B). With regard to the family level, Ruminococcaceae belonged to Firmicutes as well as Bacteroidaceae were significantly reduced in ITB compared with CD (Figures 8C,D). While 114 genera were shared by both ITB and CD, there were 34 genera and 73 genera that were exclusively found in CD and ITB, respectively (Figure 8E and Supplementary Table 4). Further statistical analysis showed a decreased abundance of genera, including *Bacteroides*, *Faecalibacterium*, and *Klebsiella*, in ITB compared with CD (Figure 8F). To explore the potential ability of the gut microbiome to discriminate ITB from CD, we used the machine learning method Random Forest to the genus level dataset. Figure 9A showed the top 20 features ordered by two specific importance measures: the mean decrease accuracy and the mean decrease in Gini. Of these, four genera (*Collinsella*, *Bacteroides*, *Faecalibacterium*, and *Klebsiella*) were chosen based on both Gini Index and relative abundance. The performance of the model was assessed using ROC analysis, and the testing results showed that *Collinsella*, *Bacteroides*, *Faecalibacterium*, and *Klebsiella* individually achieved an accuracy of 0.83 (95% CI, 0.72–0.94), 0.79 (95% CI, 0.67–0.91), 0.77 (95% CI, 0.65–0.89), and 0.57 (95% CI, 0.43–0.72), respectively (Figure 9B). Of interest, the combination of these four candidate biomarkers improved the accuracy of differentiation between ITB and CD to 0.976 (95% CI, 0.93–1) (Figure 9C).

## Discussion

Since ITB and CD share similar clinical, endoscopic, and histological features, the differentiation between these two diseases is a challenge for clinicians, especially in developing countries, where the prevalence of ITB remains high and the incidence of CD is rising (Kedia et al., 2019). Although several predictive models have been developed and validated with good accuracy, most of them involved multiple parameters, and thus, it is complicated and time-consuming for clinical application (Limsrivilai and Pausawasdi, 2021). With the development of high-throughput sequencing and bioinformatic analysis, accumulating evidence has demonstrated the great capability of the gut microbiota as a diagnostic tool for human diseases (Lopetuso et al., 2018). Herein, we delineated the community structure of mucosa-associated microbiome in patients with newly diagnosed ITB by means of 16S rRNA gene sequencing. Our data demonstrated that ITB was associated with altered composition and function of gut microbiota, including the decreased abundance of *Faecalibacterium* and *Roseburia* and the increased abundance of *Klebsiella* and *Lactobacillus*. Moreover, we found that the microbial phenotype of ITB was distinctly different from active CD, although these two intestinal diseases shared some bacterial changes in common as compared with HC. Based on the microbial signature, we established a model containing four genera that have discriminatory power for differentiating ITB from CD.

So far, numerous studies have investigated the relationship between pulmonary infection of *M. tuberculosis* and gut microbiome, yet the alterations of gut microbiota in ITB are obscure. In this study, we found that patients with ITB host a markedly different mucosa-associated gut microbiome, with a significant shift in the global diversity. The intestinal dysbiosis of ITB was defined by the dominance of Proteobacteria, which contain many pathogenic species, as well as the depletion of Firmicutes. Further differential analysis at the genus level showed that the relative abundance of *Faecalibacterium*, *Roseburia*, and *Ruminococcus*, which are recognized as the short-chain fatty acids (SCFAs)-producing microbes, was dramatically decreased in ITB. Interestingly, the reduction of SCFAs-producing bacteria was also observed in the stool samples of patients with pulmonary tuberculosis, indicating the vital role of SCFAs in the infection of *M. tuberculosis* (Luo et al., 2017; Hu et al., 2019). SCFAs, as a major group of metabolites from gut microbes, are known to exert a beneficial effect on health through regulating innate immunity and protecting gut barrier integrity (Liu et al., 2021). We speculated that the reduction of genus *Faecalibacterium* and *Roseburia* in ITB might lead to the impaired production of SCFAs and the consequent intestinal metabolic disorders. Additionally, we observed the over-growth of pathogenic *Klebsiella* and *Pseudomonas* in ITB, which may contribute to the activation of several bacterial-associated pathways as revealed by functional prediction, such as bacterial invasion of epithelial cells, lipopolysaccharide biosynthesis, and pathogenic *E. coli* infection. Taken together, the loss of beneficial microbes as well as the enrichment of opportunistic pathogen could finally promote the intestinal injury in ITB.

It is widely established that the gut microbiota plays an important role in the development of CD in both human and animal models (Pascal et al., 2017; Wu et al., 2020). In our research, the mucosa-associated microbiome of patients with active CD exhibited decreased species richness and evenness compared with HC, which agrees with previous studies (Wang et al., 2017). Low microbial diversity, which has been reported in a variety of human diseases, is considered as one of the major types of gut dysbiosis (Kriss et al., 2018). Moreover, our previous study demonstrated that the bacterial diversity in patients with active CD was significantly increased after the remission induction therapy, which suggests the association between microbial diversity and disease activity (He C. et al., 2019). Recently, several studies have reported that CD patients displayed lower relative abundances of SCFA-producing bacteria, which were correlated with the reduction of SCFAs in fecal samples (Wang et al., 2017; Wang Y. et al., 2021). In line with this finding, we also observed the decreased abundance of several SCFAs producers including *Roseburia* and *Ruminococcus* in CD relative to HC. Additionally, *Collinsella* has been identified as one of the microorganisms that could be used to discriminate CD from non-CD patients, with lower relative abundance in CD (Pascal et al., 2017). This agrees with the observation in this study.

One of the novel findings of this study was that the structure of gut microbiota in ITB and CD patients showed both common and different characteristics. Of interest, the predominant bacteria at the phylum level were Proteobacteria in both ITB and CD instead of Firmicutes in HC. Furthermore, we found that the reduction of Firmicutes in ITB and CD was attributed to the depletion of genus *Roseburia* and *Ruminococcus*, which were known as SCFAs producers. In addition to the bacterial composition, the functional analysis also showed that the pathways including the bacterial secretion system, bacteria invasion of epithelial cells, and pathogenic *E. coli* infection were significantly enriched in both ITB and CD relative to HC. We speculated that similar alterations of gut microbiota in ITB and CD might contribute to the resemblance of the endoscopic manifestations of the intestinal injury. However, there are some distinctive characteristics of gut microbiota in ITB and CD. Patients with CD exhibited lower microbial diversity relative to ITB. While the SCFAs producers including family Ruminococcaceae and genus *Faecalibacterium* were reduced in both ITB and CD as compared with HC, their abundance was much lower in ITB than CD. Moreover, we found that the widely reported SCFAs-producing bacteria such as *Faecalibacterium*, *Clostridium*, and *Bifidobacterium* were significantly increased after 6 months of antituberculosis treatment (Supplementary Figure 3). Thus, the abundance of SCFAs-producing bacteria as well as their metabolites may play a critical role in modulating immune and inflammatory response against tuberculosis, which is in concordant with previous reports (Wang S. et al., 2021).

Several limitations of this study should be noted. Although we first explored the alterations of gut microbiota in patients with ITB as well as the microbial biomarkers to differentiate ITB from CD, the number of patients was relatively small, and the predictive efficacy of bacterial candidate warrants validation in large-scale multicenter studies. Additionally, the imbalance of gut microbiota assessed by 16S rRNA gene sequencing in this study needs to be confirmed through shotgun microbiome metagenomics, which may reveal more accurate taxonomic composition and function. Finally, we investigated the changes of mucosal-associated microbiota in ITB and CD, which were probably different from fecal microbiota. As fecal samples are more readily obtained, the use of fecal bacteria as markers to distinguish ITB from CD will be determined in our future study.

## Conclusion

Our data provide a detailed description of the disruption of mucosa-associated microbiota in patients with ITB, which was characterized by the dominance of *Proteobacteria* and a dramatic loss of SCFA-producing bacteria such as *Faecalibacterium*, *Roseburia*, and *Ruminococcus*. We also observed the distinction and similarity of microbial features between ITB and CD, two intestinal diseases that are difficult to distinguish for clinicians. Biomarkers based on four mucosal bacteria can discriminate ITB from CD with good performance. Additional studies are warranted to validate the potential capability of gut microbiota as a convenient tool for the differentiation between ITB and CD.

## Data Availability Statement

The datasets presented in this study can be found in online repositories. The names of the repository/repositories and accession number(s) can be found in the article/Supplementary Material.

## Ethics Statement

The studies involving human participants were reviewed and approved by The Institutional Review Boards of The First Affiliated Hospital of Nanchang University. The patients/participants provided their written informed consent to participate in this study. Written informed consent was obtained from the individual(s) for the publication of any potentially identifiable images or data included in this article.

## Author Contributions

CH performed the bioinformatics analysis and wrote the manuscript. HW, CY, and CP collected the samples and contributed to the data collection. XS and WL performed the clinical diagnosis. ZZ designed and supervised the project. All authors contributed to the article and approved the submitted version.

## Conflict of Interest

The authors declare that the research was conducted in the absence of any commercial or financial relationships that could be construed as a potential conflict of interest.

## Footnotes

1 http://drive5.com/uchime/uchime_download.html

2 http://www.drive5.com/usearch/manual/uchime_algo.html

3 http://www.omicsmart.com

## Supplementary Material

Supplementary Figure 1 Mucosa-associated gut microbiota biomarkers for classifying patients with intestinal tuberculosis (ITB) from heathy controls (HC). Mean Decrease Gini **(A)** and Mean Decrease Accuracy **(B)** analysis showed the top 20 most important genera by the random forest test. **(C)** The accuracy of candidate biomarkers was verified with cross-validation. The area under curve (AUC) value was calculated and receiver operating characteristic curves (ROC) were drawn with five repeats.

Supplementary Figure 2 Identification of the gut microbial signatureassociated with Crohn’s disease (CD). The top 36 most important genera were assessed by the Mean Decrease of Gini **(A)** andaccuracy **(B)**. **(C)** The ROC curve of the optimal model containing 3 genera for classifying CD from healthy controls (HC).

Supplementary Figure 3 The ITB-diminished genera were significantly increased after 6 months of anti-tuberculosis treatment. One of the ITB patients was followed up and the intestinal ulcer was completely healed after the quadruple regimen (isoniazide, rifampicin, pyrazinamide, ethambutol) for 6 months. The mucosal samples from ileum, ascending colon and descending colon were collected before and after treatment and then the changes of gut microbiota were compared at genus level by Welch’s *t* test.
