# Supplementary figures and images for: Alterations of Gut Microbiota in Patients With Intestinal Tuberculosis That Different From Crohn’s Disease
Source: Front Bioeng Biotechnol. 2021 Jul 6;9:673691. doi: 10.3389/fbioe.2021.673691 (PMC8290844; doi:10.3389/fbioe.2021.673691)

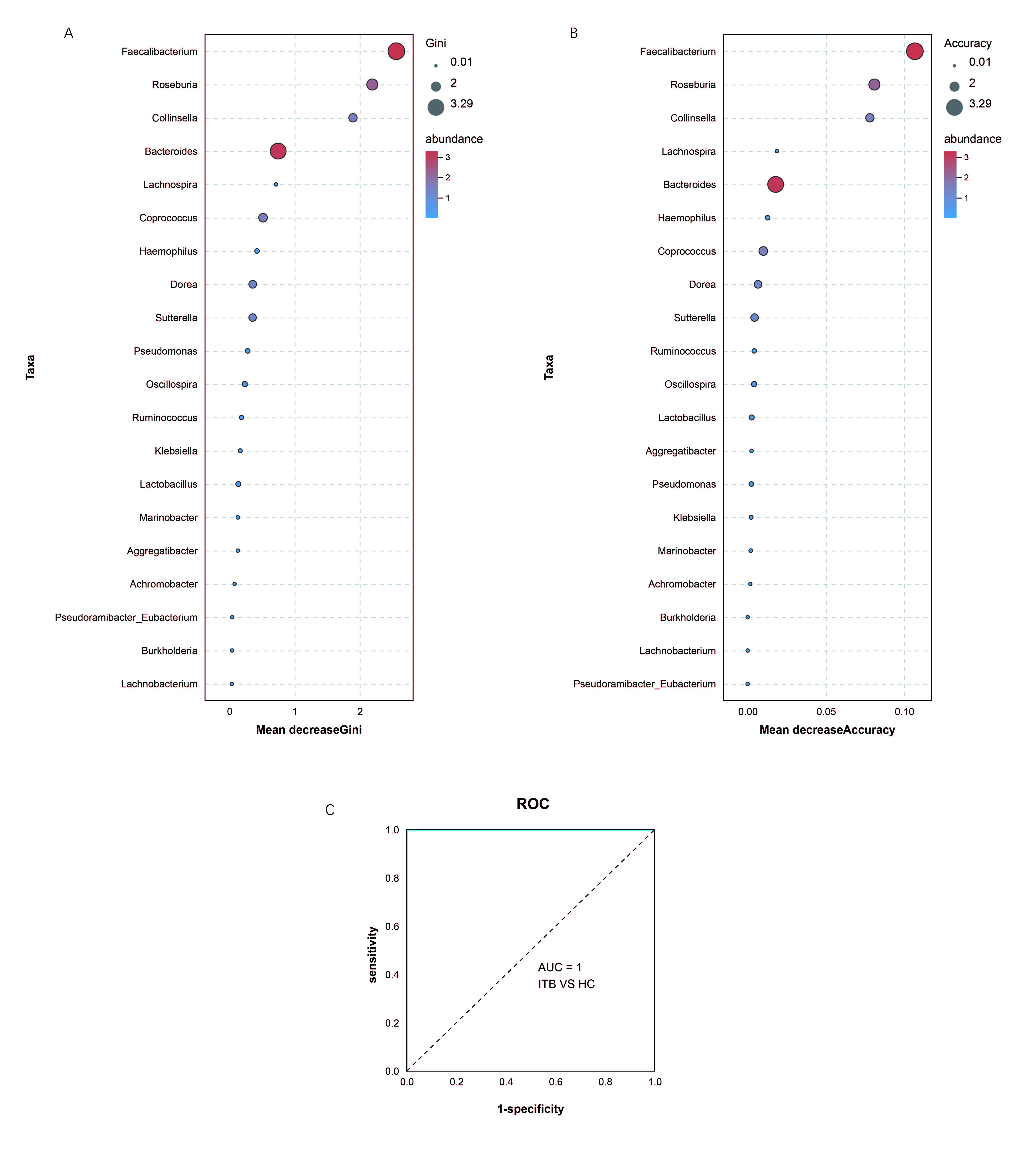

Supplement: Supplementary Figure 1 — Mucosa-associated gut microbiota biomarkers for classifying patients with intestinal tuberculosis (ITB) from heathy controls (HC). Mean Decrease Gini (A) and Mean Decrease Accuracy (B) analysis showed the top 20 most important genera by the random forest test. (C) The accuracy of candidate biomarkers was verified with cross-validation. The area under curve (AUC) value was calculated and receiver operating characteristic curves (ROC) were drawn with five repeats. [file Image_1.JPEG]

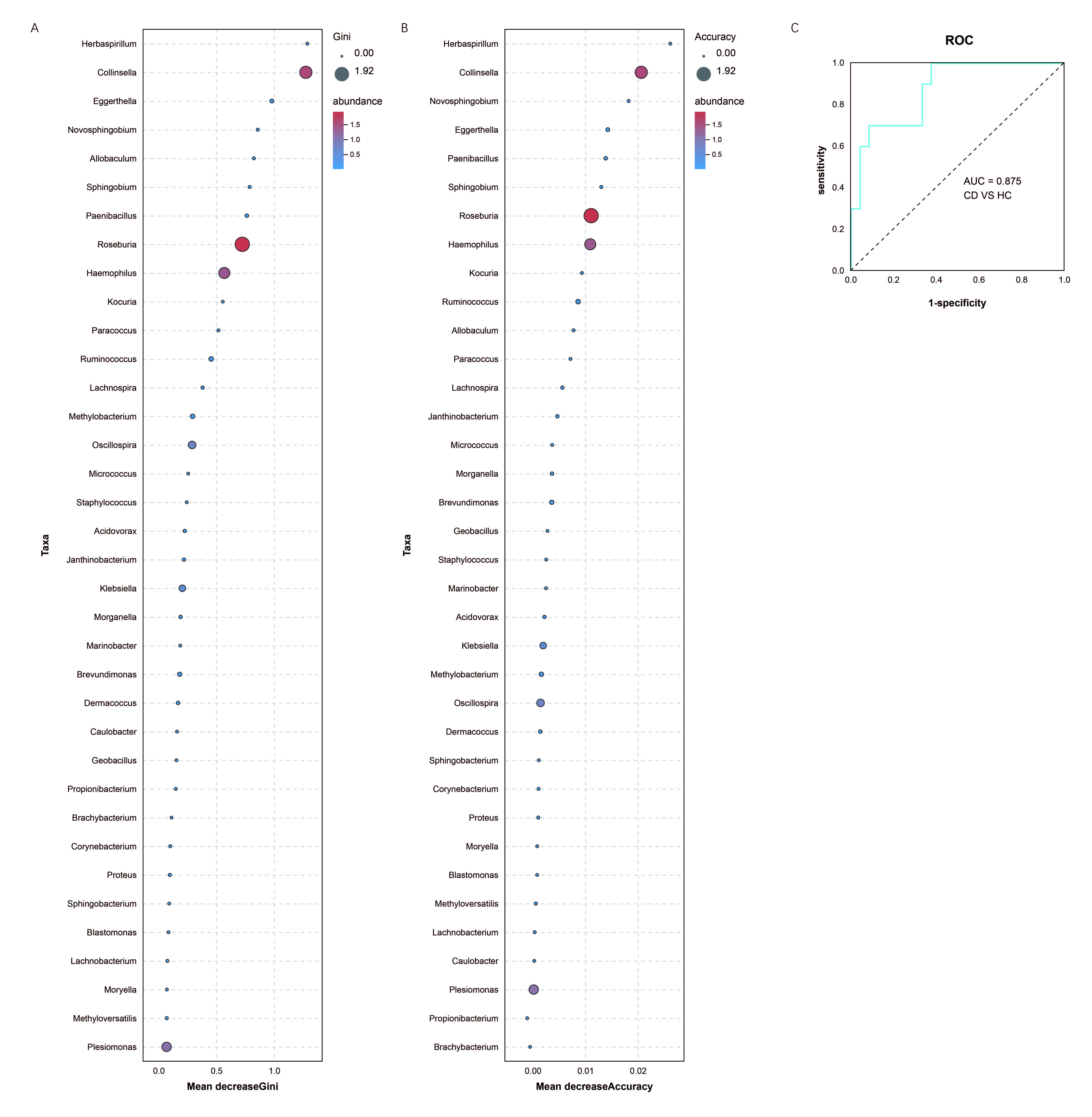

Supplement: Supplementary Figure 2 — Identification of the gut microbial signatureassociated with Crohn’s disease (CD). The top 36 most important genera were assessed by the Mean Decrease of Gini (A) andaccuracy (B). (C) The ROC curve of the optimal model containing 3 genera for classifying CD from healthy controls (HC). [file Image_2.JPEG]

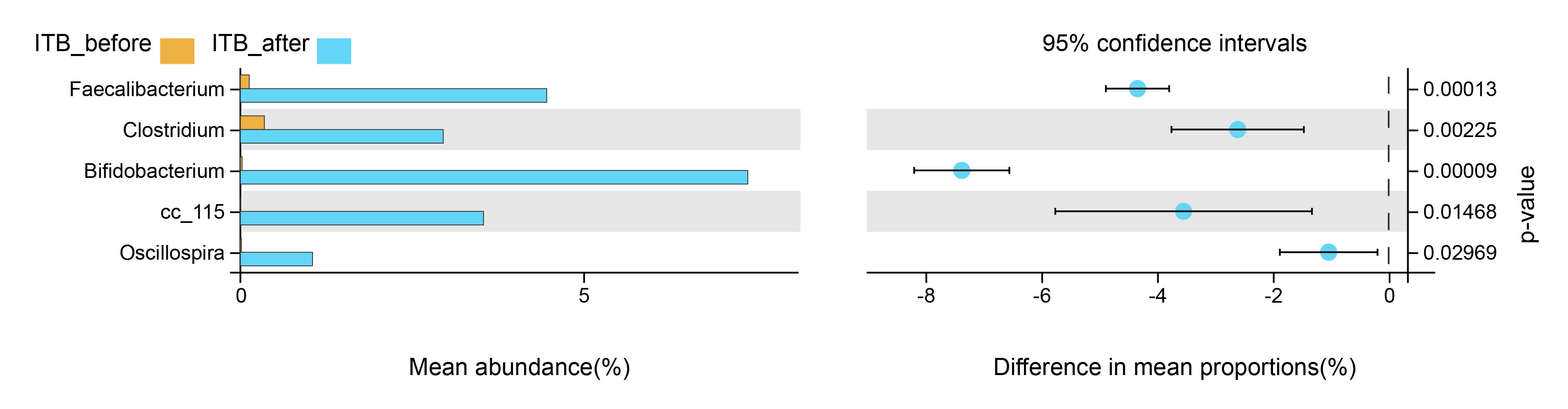

Supplement: Supplementary Figure 3 — The ITB-diminished genera were significantly increased after 6 months of anti-tuberculosis treatment. One of the ITB patients was followed up and the intestinal ulcer was completely healed after the quadruple regimen (isoniazide, rifampicin, pyrazinamide, ethambutol) for 6 months. The mucosal samples from ileum, ascending colon and descending colon were collected before and after treatment and then the changes of gut microbiota were compared at genus level by Welch’s t test. [file Image_3.JPEG]
